# Incidental Diagnosis of Duodenal Giardiasis

**DOI:** 10.7759/cureus.15499

**Published:** 2021-06-07

**Authors:** M Ammar Kalas, Ahmed Alduaij, Amer A Alkhatib

**Affiliations:** 1 Internal Medicine, Texas Tech University Health Sciences Center El Paso, El Paso, USA; 2 Pathology and Laboratory Medicine, Cleveland Clinic Abu Dhabi, Abu Dhabi, ARE; 3 Gastroenterology and Hepatology, Cleveland Clinic Abu Dhabi, Abu Dhabi, ARE

**Keywords:** giardia lamblia, malabsorption, giardiasis, endoscopy, giardiasis diagnosis

## Abstract

*Giardia lamblia* is a protozoan that results in the commonly diagnosed giardiasis. Due to its prevalence and incidence worldwide, it is essential to recognize the different presentations of giardiasis, routes of transmission, modalities of diagnosis, treatments, and complications. Diagnostic modalities have evolved over the years and can be achieved reliably through noninvasive stool testing. Endoscopic duodenal aspirate and biopsies can also be used; however, they yield lower sensitivity and specificity rates and are therefore not used as a first-line modality for diagnosing giardiasis. Nonetheless, its use should be considered in cases with high suspicion and unremarkable stool testing. Herein, we discuss the case of a 27-year-old male with chronic weight loss and abdominal pain for six months diagnosed with *G. lamblia* through a duodenal biopsy.

## Introduction

Giardiasis is one of the most common intestinal infections worldwide in both developed and developing countries. It has an estimated incidence of approximately 280 million cases per year globally and more than one million cases annually in the United States [1]. The protozoan *Giardia lamblia* generally causes giardiasis; however, there are other *Giardia* species that are not addressed in this case report. *Giardia* is a kite-like protozoan parasite that infects the proximal small intestine. It leads to sporadic and endemic diarrhea. It has multiple modes of transmission such as foodborne, waterborne, and can even be sexually transmitted via the fecal-oral route. The protozoa have two forms, the cyst and the trophozoite. Once the cysts are ingested, excystation occurs in the duodenum, resulting in the release of trophozoites. The trophozoites have flagellar structures that attach to the proximal intestinal mucosa. In the mucosa, some become cysts, which is the infective form, and pass out with feces [2]. Outside the host, cysts can survive up to three months.

## Case presentation

**Figure 1:**
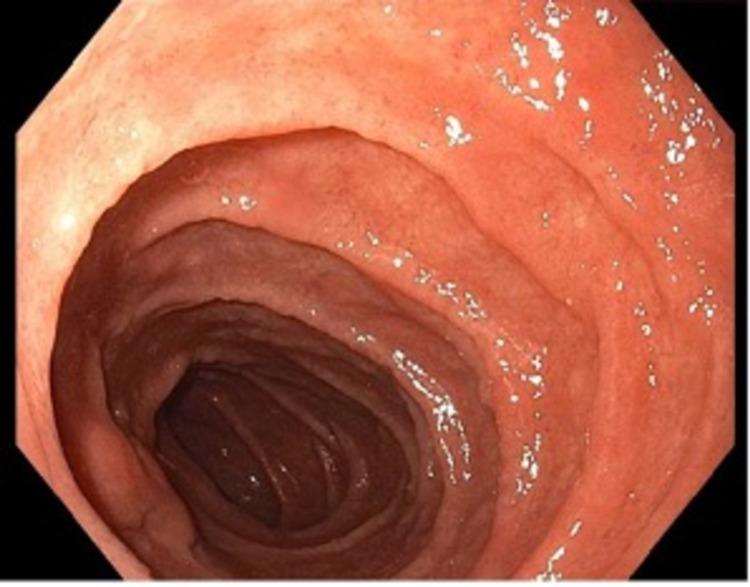
Congested duodenal mucosa on upper endoscopy.

**Figure 2:**
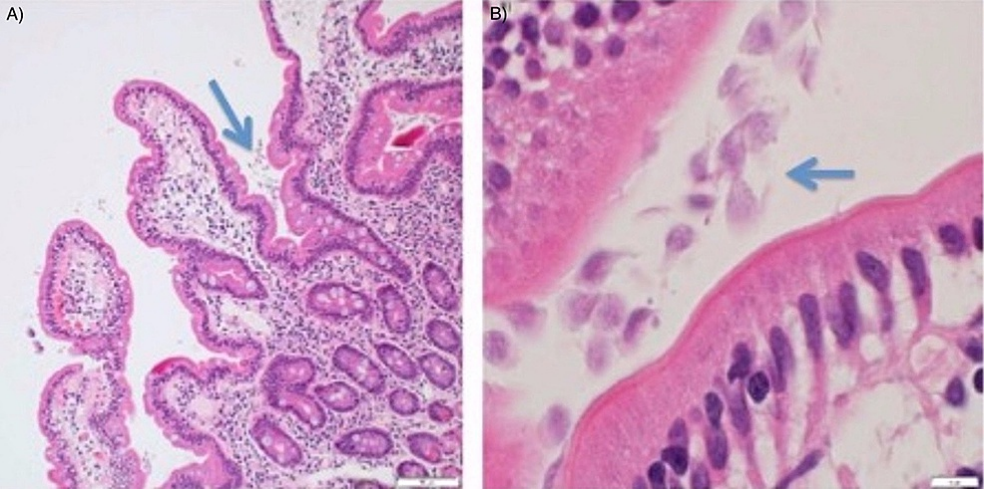
Duodenal biopsy images. (A) Duodenal biopsy with numerous *Giardia *organisms visible along the villous surface (H&E, 100×). (B) Duodenal biopsy with numerous parasites featuring double nuclei compatible with *Giardia lamblia* around the intestinal villi (H&E, 1000×). H&E: hematoxylin and eosin

A 27-year-old male presented to the clinic with a six-month history of epigastric abdominal pain. The pain was intermittent, nonradiating, unrelated to oral intake, and burning in nature with no alleviating or aggravating factors. Moreover, the patient reported weight loss without loss of appetite. He denied recent use of nonsteroidal anti-inflammatory medications, bowel habit changes, steatorrhea, history of recent travel, hiking, or contacts with other individuals with similar symptoms. On examination, no abdominal tenderness, distension, or masses were noted, and his bowel sounds were normal and heard in the four quadrants. Labs showed a hemoglobin level of 16.9 mg/dL and a total leukocyte count of 12,700/mm^3^. In addition, liver enzymes and kidney function tests were within normal limits. Stool antigen assays and nucleic amplification tests (NAATs) were deferred due to the absence of bowel habit changes. *Helicobacter pylori* stool antigen testing was negative. Upper endoscopy was performed and showed congestion of the duodenal mucosa (Figure 1). Therefore, duodenal biopsies were obtained which showed numerous parasites featuring double nuclei compatible with *G. lamblia* around the intestinal villi (Figure 2). The patient was treated with metronidazole as an outpatient with the resolution of symptoms over the following one month.

## Discussion

Giardiasis is an infection that can present variably; patients can be asymptomatic (carriers), acutely infected, or chronically infected. Asymptomatic patients can have shedding of the organism for up to six months and hence are infective. It was observed that in children under the age of two years, asymptomatic giardial infection was associated with growth stunting [3].

The presentation of acute infection is variable with the most common presenting symptoms being diarrhea, fatigue, steatorrhea, and abdominal discomfort. Giardiasis generally has an incubation period of one to two weeks, and symptoms occurring within one week are generally due to other causes [4]. The presentation of chronic infection can be tricky as patients can develop a chronic infection without a preceding acute illness, resulting in misdiagnosis or lack of testing for giardial infection. The presenting symptoms of chronic giardiasis generally include steatorrhea, abdominal bloating, weight loss, failure to thrive, vitamin deficiencies, and malabsorption, and approximately 40% develop reversible lactose intolerance [5].

The diagnosis of giardial infection is generally achieved with stool antigen assays (direct immunofluorescent assays), which detect cyst or trophozoite antigens in the stool using *Giardia*-specific antibodies. Studies have shown higher detection rates with antigen assays than stool microscopy with an estimated 12% discrepancy [6]. NAATs have also been developed for giardial infection and are also commonly used for giardiasis infection diagnosis. The cost of antigen assays and NAATs is comparable and both are used for infection detection [7].

Upper endoscopy use for giardial infection has not been well studied owing to the high specificity and sensitivity of noninvasive testing such as stool antigen assays. A study compared the sensitivity and specificity of endoscopy and stool antigen assays and found a significant number of false-negative results in patients tested using endoscopy compared to stool testing. Interestingly, colonoscopy was found to have a better rate of detection of giardial infection compared to upper endoscopy [8]. Hence, endoscopic diagnosis remains of lower importance when testing for giardial infection. However, in cases of negative stool testing, endoscopy would be warranted to rule out malabsorption syndromes in addition to identifying *G. lamblia* on biopsy [9].

Duodenal aspirate examination can also be performed under microscopy to detect the presence of *Giardia *cysts or trophozoites. However, the duodenal aspirate examination can be foregone as biopsies were found to have a better detection rate than aspirate microscopic examination [8]. In contrast, a prospective study done in 2003 showed no difference between duodenal biopsy and duodenal aspirate in detection of giardial infection; however, a recommendation of biopsy usage was made due to cost [10].

Treatment for giardial infection involves correction of electrolyte imbalances and vitamin deficiencies along with initiation of antimicrobial therapy. First-line antimicrobial therapies include tinidazole and nitazoxanide. Tinidazole has the advantage of single-dose administration (2 g orally) and a better side effect profile compared to metronidazole. Nitazoxanide dosing (age over four years) is 500 mg orally twice a day for three days [11]. Alternative agents include metronidazole, mebendazole, albendazole, paromomycin, furazolidone, and quinacrine. Due to a lack of studies including patients less than 12 months of age, metronidazole is the commonly used agent. Following treatment, resolution of acute infection generally occurs within one week; however, it can take longer in patients with chronic giardiasis where symptoms can linger for months. Patients with recurrence of symptoms following a symptom-free interval can develop reinfection and can be treated with any first-line agent. However, for patients with minimal improvement in symptoms after the initial treatment, repeat treatment should be attempted with a medication from a different class. Failure of initial treatment with a nitroimidazole such as tinidazole or metronidazole can be treated with a course of nitazoxanide instead [12].

Finally, patients with refractory infection will likely require combination therapy. Nonetheless, combination regimens have not been studied extensively. Commonly used combinations include but are not limited to albendazole with metronidazole, tinidazole with quinacrine, or tinidazole with metronidazole [13].

## Conclusions

Duodenal giardiasis is a common intestinal infection with a variable presentation as it can be asymptomatic, acute, or chronic. High suspicion for this infection should be considered in patients with chronic weight loss, specifically in endemic areas and developing countries. Noninvasive methods of diagnosis are very sensitive and specific. However, endoscopic biopsies can be useful in cases with high suspicion and negative stool testing, in part to diagnose giardiasis and rule out other malabsorption causes.
